# Extracellular matrix macromolecules: potential tools and targets in cancer gene therapy

**DOI:** 10.1186/2052-8426-2-14

**Published:** 2014-05-02

**Authors:** Annele Sainio, Hannu Järveläinen

**Affiliations:** Department of Medical Biochemistry and Genetics, University of Turku, Turku, Finland; Division of Endocrinology, Department of Medicine, Turku University Hospital, Kiinamyllynkatu 4-8, Fl-20520 Turku, Finland

**Keywords:** Extracellular matrix, Macromolecules, Tumour microenvironment, Cancer, Gene therapy

## Abstract

Tumour cells create their own microenvironment where they closely interact with a variety of soluble and non-soluble molecules, different cells and numerous other components within the extracellular matrix (ECM). Interaction between tumour cells and the ECM is bidirectional leading to either progression or inhibition of tumourigenesis. Therefore, development of novel therapies targeted primarily to tumour microenvironment (TME) is highly rational. Here, we give a short overview of different macromolecules of the ECM and introduce mechanisms whereby they contribute to tumourigenesis within the TME. Furthermore, we present examples of individual ECM macromolecules as regulators of cell behaviour during tumourigenesis. Finally, we focus on novel strategies of using ECM macromolecules as tools or targets in cancer gene therapy in the future.

## Introduction

Normally, the extracellular matrix (ECM) is composed of a dynamic 3D network of macromolecules, particularly collagens, elastin, proteoglycans (PGs) and hyaluronan (HA), and other non-collagenous matrix glycoproteins [1, 2]. In the ECM there are also ECM degrading enzymes as well as a variety of soluble factors such as growth factors, chemokines and cytokines [1]. Furthermore, within the ECM there are several cell types including fibroblasts, adipocytes, epithelial and endothelial cells as well as different immune cells [3]. The principal function of the ECM is to maintain normal architecture and homeostasis of a particular tissue. The composition of the ECM is unique to each tissue and it undergoes constant enzymatic and non-enzymatic modifications and remodeling processes through a biophysical dialogue between its components [4]. These modifications and remodeling processes result in versatile microenvironments, “niches”, which in turn vitally regulate the behaviour of the cells within the ECM [5].

In cancer, the malignant cells are known to create their own tumour microenvironment (TME) which crucially affects both the malignant cells themselves and all other cells within the ECM [5–8]. As tumours are composed of a mixture of different cells, the effect of TME on the malignant cells can vary depending on the cell type in question. For example, cancer stem cells (CSCs), which usually form a small portion of the whole tumour, can create their own “CSC niche” within the TME which then regulates their proliferation and also causes a barrier to anticancer therapeutics [9]. Besides CSCs, in the tumours there are also several other cell types like cancer-associated fibroblasts (CAFs), tumour associated macrophages (TAMs) and neutrophils (TANs). The presence of inflammatory cells emphasizes the importance of inflammation in tumourigenesis [3]. Regarding TAMs, two subtypes, namely M1 (tumour preventing) and M2 (tumour promoting) have been recognized [10]. Similarly, TANs have been shown to exhibit two separate phenotypes, N1 (phenotype with antitumoural properties) and N2 (protumoural phenotype) [11]. These cells represent the double role of autoimmunity with both pro- and antitumoural effects [12]. The above mentioned cells together with CAFs are able to variously secrete ECM macromolecules (e.g., collagen type I, biglycan, versican, fibronectin) as well as growth factors and cytokines [e.g., vascular endothelial growth factor (VEGF), tumour necrosis factor α (TNF-α) and interleukin 6 (IL-6)] contributing to tumourigenesis [13–17].

Apart from the ECM macromolecules, growth factors and cytokines mentioned above, there are other essential groups of molecules regulating tumour initiation and progression. For example, overexpression of ECM degrading enzymes such as matrix metalloproteinases (MMPs) can promote tumourigenesis. Indeed, in ovarian cancer the expression of MMP-2 and MMP-9 has been shown to correlate with poor survival indicating increased disseminating capability of cancer cells [18, 19]. In addition to MMPs, other members of the metzincin superfamily such as a disintegrin and metalloproteinases (ADAM) and ADAM with thrombospondin motifs (ADAMTS) are known to be critically involved in ECM turnover and remodeling during tumourigenesis [20–22]. The same applies to the family of lysyl oxidase (LOX) enzymes and transglutaminases that also represent central molecules in regulating ECM organization and tumour progression [23, 24]. Furthermore, cell membrane adhesion molecules called integrins are importantly involved in the development of tumours. For example, in prostate cancer metastasis integrin αvβ6 expression has been shown to induce the expression of MMP-2 which in turn mediates osteolysis via its matrix degrading activity [25]. Certainly, other molecules and mechanisms whereby TME is involved in tumourigenesis could be presented [5, 26, 27].

Tumour progression also requires angiogenesis. Normally angiogenesis is strictly regulated. However, in the TME various cells can overexpress angiogenesis stimulating growth factors like VEGF ensuring oxygen and nutrient delivery to the growing tumour cell mass [28, 29]. In addition to certain growth factors, specific ECM macromolecules by themselves are known to be able to regulate angiogenesis [30]. As the central role of the ECM and its components in tumourigenesis has become a recognized fact, to study the regulatory functions of the ECM macromolecules in the TME is of vital importance [31]. In this review we will focus on the ECM macromolecules in cancer and on the possibility to exploit them as novel therapeutic tools or targets in cancer gene therapy.

## Review

### ECM macromolecules and cancer

As mentioned in the beginning, ECM macromolecules can be categorized into four main groups: collagens, elastin and microfibrillar proteins, PGs and HA, and other non-collagenous matrix glycoproteins [2]. From these main groups, a subclass of secreted proteins, so-called “matricellular proteins” can be segregated [32–34]. They comprise a group of ECM macromolecules including thrombospondin- 1 and −2, SPARC (secreted protein, acidic and rich in cysteine), tenascin C (TN-C) and osteopontin [34]. The matricellular proteins do not directly participate in the formation of structural elements but are rather involved in the modulation of cell-matrix interactions and cell function. For example, TN-C is often associated with increased invasiveness of tumour cells [35]. On the basis of the ability of individual ECM macromolecules to form fibers, classification into fiber-forming and interfibrillary matrix molecules can also be made. Most of these ECM macromolecules have been shown to be variously associated with cancer as described below.

Collagen is the most prominent structural protein of the ECM and 28 collagen types have been identified [36]. Different collagens, particularly types I and III are often associated with cancers such as breast and pancreatic cancers resulting in increased stromal collagen accumulation and promotion of cancer progression [37–40]. In breast cancer, collagen accumulation together with increased expression of small leucine-rich proteoglycans (SLRPs) decorin and lumican has been shown to correlate with increased mammographic density [41]. Also degradation of specific collagens has been observed during metastasis and the resulting collagen fragments can recruit e.g. TAMs whose abundance in tumours predicts poor prognosis [39, 42]. On the other hand, degradation of collagen type XVIII resulting in the formation of endostatin causes inhibition of angiogenesis and thereby retards tumour growth [43]. Furthermore, some collagens such as type XXII and XXIV have been referred to have prognostic value in certain cancers [44].

Similarly to the richness of collagen types, over 30 mostly extracellular PG species have been identified [45, 46]. With few exceptions PGs comprise a protein core to which one or more glycosaminoglycan (GAG) side chains are covalently linked. Many of the PGs, like certain family members of the SLRPs have been shown to be involved in the organization of the ECM [47]. For example, the SLRPs decorin, biglycan, fibromodulin and lumican are centrally involved in the regulation of collagen fibrillogenesis [48–51]. The above mentioned SLRPs are also variously involved in tumourigenesis. Especially decorin has a recognized regulatory role in tumour development, most notably via its ability to down regulate several members of the receptor tyrosine kinase (RTK) family members such as the epidermal growth factor receptor (EGFR) [52–54]. Furthermore, decorin can regulate the activity of the Met receptor, i.e., the receptor for hepatocyte growth factor, and the insulin-like growth factor receptor I (IGF-IR) [55, 56]. Interestingly, decorin can also regulate tumour angiogenesis by reducing the production of e.g., VEGF [57, 58]. Other mechanisms whereby decorin contributes to tumour growth will be discussed later on. Regarding lumican, it has been shown to variously modulate proliferation, migration and adhesion of cancer cells. For example, inhibition of tumour cell migration by lumican has been shown to be mediated via its interaction with α2β1 integrin [59]. Similarly to decorin, lumican can also regulate cancer associated angiogenesis [60].

Hyaluronan is a versatile non-sulfated GAG that consists of repeating D-glucuronic acid and N-acetyl-D-glucosamine disaccharides. It has a widely recognized tumour promoting role in several cancer types such as prostate, ovarian and breast cancers via activating e.g., CD44 and RHAMM (receptor for hyaluronan-mediated motility) mediated signaling pathways including NFκB and mitogen-activated protein kinase (MAPK) pathways [61–63]. Indeed, its stromal accumulation is typical of progressed and undifferentiated tumours predicting poor prognosis [64–66].

Non-collagenous matrix glycoproteins like fibronectin have also been linked with cancers such as lung cancer, where its overexpression correlates with invasive and metastatic phenotype [67]. Fibronectin can bind to other ECM molecules and integrins including α_v_β_3_ and thereby regulate among its other functions cell adhesion and Epithelia-Mesenchymal Transition (EMT) [68]. Different ECM molecules may also exist as tumour promoting splice variants as has been described e.g. for TN-C in breast cancer [69].

En bloc, ECM macromolecules are of central importance in cellular biology and they must act in concert in a finely regulated manner to maintain homeostasis and cellular functions within tissues and organs [70]. When the ECM is affected by inherited defects or its components are dysregulated, the proper 3D network of the matrix is lost and the cell-matrix interactions are hampered leading to or enabling different disease processes. Below, we will present some mechanisms whereby ECM macromolecules promote or alternatively inhibit tumourigenesis.

### ECM macromolecules as regulators of tumourigenesis within the TME

Remodeling of the ECM is considered one of the earliest steps in the formation of TME which involves most ECM proteins [71]. Today we understand that there is an interaction between ECM macromolecules and cancer cells within each specific TME that can either facilitate or counteract the growth of solid tumours [9, 72, 73]. The TME can also restrict drug delivery to malignant cells [3, 9]. As different ECM molecules possess central functional roles in various cellular events crucial in tumourigenesis like proliferation, adhesion, migration, survival and differentiation of the cells, they represent potential and novel targets and tools for pharmacotherapy [2].

With the help of the TME, cancer cells are able to exploit various mechanisms that can increase their malignancy and survival, e.g. they use autophagy to survive in nutrient-limited and hypoxic microenvironments [74]. On the other hand, autophagy has also been linked to suppression of tumourigenesis through elimination of p62 in tumour cells [75]. Interestingly, the SLRP decorin has been found to act as a regulator of endothelial cell autophagy which results in the inhibition of angiogenesis and suppression of tumour growth [76]. More precisely, soluble decorin induces the expression of Peg3, a transcription factor usually silenced in cancer and causes inhibition of endothelial tubulogenesis and cell migration [77]. In addition to regulating autophagy, decorin is able to cause mitostatin-dependent mitophagy by inducing the expression of mitostatin via the Met receptor (the receptor for hepatocyte growth factor) resulting in concurrent decrease in VEGF-A transcription in breast cancer cells and subsequently inhibition of angiogenesis [78].

Other mechanisms that can be exploited by cancer cells via ECM macromolecules are Epithelia-Mesenchymal Transition (EMT) and its reverse process called Mesenchymal-Epithelial Transition (MET) [79]. The epithelial plasticity is a central part of normal development, but its regulators including *myc*, growth factors such as TGF-β, and β-catenin can be misexpressed in cancers like breast [80], ovarian [81] and colon [82] cancers, respectively. This plasticity provides tumour cells with phenotypes which enable them to escape from the primary tumour. As breast cancer cells among other malignant cell types are known to secrete TN-C, an ECM macromolecule often associated with the invasive front of tumours [83, 84], its expression together with the production of TGF-β1 can cause the malignant cells to undergo EMT [85]. Furthermore, integrins αvβ1 and αvβ6 are able to independently participate in this TN-C-induced EMT-like change [86]. The EMT also involves several other molecules including cadherins, focal adhesion molecules and proteolytic enzymes, e.g. MMPs [87]. All the above mechanistical possibilities have to be taken into account when novel therapies focusing on the ECM are developed.

### ECM macromolecules as tools or targets in gene therapy

As discussed above, marked quantitative changes in the composition of the ECM macromolecules are a typical feature of different types of cancer. However, the distinction must be made whether these changes are the cause of the malignant disease or the result of it [7]. In spite of this, because individual ECM macromolecules can significantly influence cell behaviour and disease processes, modulation of the ECM composition provides a rational way to be used as an optional novel therapy in diseases like cancer. This modulation could be achieved with a number of approaches including gene therapy where specific ECM macromolecules are used as tools or targets.

#### Challenges using gene therapy

Generally, the development of gene therapy sets several challenges [88]. These challenges include choice of the vector to deliver the gene, the delivered gene itself and the route of administration. Furthermore, viruses must target diseased cells with enough large numbers to achieve wanted effects and at the same time with minimal destruction of the normal cells [89, 90]. Timing of the gene delivery is also a critical issue to be considered because several tumours undergo marked temporal changes, as can be seen e.g. during the initiation and progression of pancreatic cancer [91]. Some tumour types like pancreatic cancer can also contain vast amounts of HA resulting in exceptionally high interstitial fluid pressure in solid tumour tissue, which in turn can prevent perfusion and diffusion of small molecule therapeutics [92]. In addition to the above challenges, alterations in the chemomechanical environment of cancer cells has to be recognized [5, 93–95].

When gene therapy for cancer using or targeting ECM macromolecules is developed, the challenges can become even more sophisticated. For example, regarding the timing of gene therapy it is noteworthy to take into account that collagen expression has been discovered to have a dual role in tumour progression; different collagen types can either restrict or promote tumour development depending on the stage of the tumour [6, 96]. Furthermore, phenomenon known as desmoplastic reaction, i.e., the formation of a sclerotic stroma around the cancer cell population due to the overexpression of certain collagen types can make the gene targeting problematic [7]. This dense fibrotic deposition is typical of cancers like colorectal [97, 98], pancreatic [99, 100] and liver [101] cancers. In addition to the possible increased amount of ECM macromolecules around the cancer cells, most cancers are of epithelial origin and still maintain intercellular junctions despite their de-differentiated nature further restricting gene targeting [102]. However, it is possible to overcome these challenges as is discussed in the following two sections. Thus, gene therapy may have a promising future.

#### Strategies to overcome ECM macromolecule formed barrier in cancer treatment using gene delivery

Excess accumulation of the ECM macromolecules in the TME plays a critical role in blocking the transport of therapeutics to the target cells. Specifically, fibrillar collagen and collagen-proteoglycan bonds can form a major limit to the delivery of gene vectors [103, 104]. Even nanomedicine utilizing nanoparticles can confront this same problem [105]. Regarding drug penetration to the tumour, the use of ECM macromolecules in gene therapy could provide a solution. For example the use of decorin containing replicating oncolytic adenovirus can increase drug penetration to solid tumours leading to a dramatic anti-tumour effect [102]. This beneficial effect of decorin gene delivery has been suggested to be based on decreased expression of other ECM molecules within the tumour tissue [102]. The physical barrier formed by ECM macromolecules is also possible to overcome by the administration of hyaluronidase (HAase) or collagenase containing oncolytic adenoviruses, which have been found to lead to significant tumour regression [106–108]. Additionally, the use of HAase can enhance the action of immune effector cells via degradation of the HA formed halos around cancer cells like adherent fibrosarcoma cells [109]. Treatment with HAase has also been shown to stimulate hematopoiesis and to increase the number of neutrophils in the peripheral blood [110].

Applying the same ECM degrading principle with other enzymes such as MMPs can be valuable [111]. For example, adenoviral expression of MMP-8, which breaks down collagen type I, II, and III, is a potential strategy for improving ECM hindered drug penetration or viral spread [112]. Apart from degrading excess ECM in the tumour matrix, reducing ECM production or modifying ECM organization by means of pharmacological intervention offers potential strategies to improve drug penetration [113, 114]. This kind of manipulation of the tumour matrix structure can be considered as “matrix normalization” [105, 113, 114].

#### Inhibition of tumour progression using selected ECM macromolecules via gene delivery

**Figure 1:**
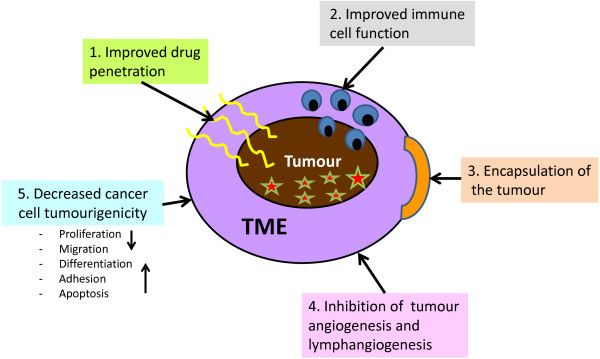
**Schematic representation of the desired effects of ECM-based gene therapies in cancer. 1**. Degradation or reorganization of ECM macromolecules in the TME enables more efficient drug delivery to the tumour. **2**. Degradation of ECM macromolecules, e.g. degradation of HA formed halos around cancer cells can enhance action of immune cells. **3**. Encapsulation of the tumour mass with specific ECM macromolecules could inhibit tumour growth and metastasis. **4**. Diminished tumour angiogenesis and lympangiogenesis can cause tumour necrosis and prevent metastasis. **5**. Manipulation of 3D structure of the ECM in TME can decrease cancer cell tumourigenesis via various mechanisms. TME: tumour microenvironment.

A myriad number of ECM macromolecules are known to be involved directly or indirectly in tumourigenesis [5]. This provides several possibilities for using ECM macromolecules as tools in gene therapy. For example, expression of human decorin cDNA via adenovirus mediated transfection has been demonstrated to induce specific and even distant apoptosis of cancer cells demonstrating a direct antitumourigenic effect of decorin [115, 116]. In addition to decorin, there are other PGs such as lumican and its core protein called lumcorin that have been found to possess antitumourigenic functions and could therefore be used as a chosen molecule in gene therapy [117–119]. Furthermore, human fibronectin containing recombinant adenovirus has been shown to be a promising strategy as a novel gene therapy against metastatic breast cancer via its inhibitory effect on adhesion of cancer cells to ECM molecules [120]. Additional ECM-based gene therapy strategies also exist. Regarding HA, interactions between HA and its major cell surface receptor, CD44, have raised interests on the basis of influencing on CD44 variants (CD44v) of cancer cells [121]. For example, cell-specific delivery of shRNA (short hairpin RNA) targeting HA-CD44v6 has been shown to lead to marked inhibition of tumour growth in mice [122]. In the case of collagen, particularly the 20 kDa C-terminal cleavage product of collagen type XVIII (endostatin) provides a very potent candidate in gene therapy in the future [43]. Endostatin is able to markedly inhibit not only angiogenesis but also lymphangiogenesis [123]. Adenoviral transfection carrying endostatin to bladder cancer cells in mouse models has already been shown to significantly decrease tumour progression via angiogenesis inhibition [124]. In theory, gene delivery of specific ECM macromolecules such as collagen and decorin could also be applied to encapsulate the tumour mass, thus possibly restricting tumour growth and metastasis [7, 58]. A summary of the desired effects of different ECM-based gene therapies on tumours is presented in Figure 1.

## Conclusions

The ECM has a recognized role in the regulation of key cellular functions such as adhesion, proliferation, migration and apoptosis. Also the differentiation of normal and cancer associated cells is known to be highly dependent on the interaction of the cells with their microenvironment. Therefore, the ECM is critically involved in various disease processes including tumourigenesis where significant changes in the composition and structure of the ECM can be observed. Although it has not been clearly established whether the observed ECM changes are the cause or the result of various diseases including cancer, strategies to modulate the structure of the ECM offers therapeutical possibilities. These could be achieved e.g. via gene therapy utilizing specific ECM macromolecules as tools and/or targets. As discussed in this review, the current literature offers solid rationale to the development of gene therapies focusing primarily on the ECM. However, several challenges must still be resolved. These include choice of the vector and the delivered gene itself, timing of the delivery and route of administration. Nevertheless, ECM-based gene therapies form a very attractive and promising field of research that is likely to advance novel treatments of cancer in the future.

## Abbreviations

ADAM: A disintegrin and metalloproteinases
ADAMTS: A disintegrin and metalloproteinases (ADAM) with thrombospondin motifs
CAF: Cancer-associated fibroblast
CD44v: CD44 variants
CSC: Cancer stem cells
ECM: Extracellular matrix
EGFR: Epidermal growth factor receptor
EMT: Epithelial-mesenchymal transition
GAG: Glycosaminoglycan
HA: Hyaluronan
HAase: Hyaluronidase
IGF-IR: Insulin-like growth factor receptor I
IL-6: Interleukin 6
MAPK: Mitogen-activated protein kinase
MET: Mesenchymal-epithelial transition
Met: Mesenchymal-epithelial transition [Met] receptor/receptor for hepatocyte growth factor
MMP: Matrix metalloproteinase
PG: Proteoglycan
RHAMM: Receptor for hyaluronan-mediated motility
RTK: Receptor tyrosine kinase
shRNA: short hairpin RNA
SLRP: Small leucine-rich proteoglycan
SPARC: Secreted protein, acidic and rich in cysteine
TAM: Tumour-associated macrophage
TGF-β: Transforming growth factor beta
TME: Tumour microenvironment
TN-C: Tenascin
TNF-α: Tumour necrosis factor α
VEGF: Vascular endothelial growth factor.
